# 

**Published:** 1811-08

**Authors:** 


### MONTHLY CATALOGUE OF MEDICAL BOOKS.

Cheselden's Plates of the Human Bones, correctly reduced from the original copy, accompanied with explanations. 12mo. Cox.

An Account of the Ravages committed in Ceylon by the Small Pox, previously to the Introduction of Vaccination: with a Statement of the Circumstances attending the Introduction, Progress, and Success of Vaccine Inoculation in that Island. By Thomas Christie, M. D. 8vo. Murray.

History

History of the Walcheren Remittent; commencing with its advanced state, when most dangerous and destructive to the Soldiery, and concluding with its very favourable Termination, effected by those means first proposed by the Author only to the Legislature, and to the late and present Army Medical Board, with the Morbid Appearances on Dissection. Also the Sequels, Anæmia acholia, Egyptian Ophthalmia, &c. &c. &c. By Thomas Wright, M. D. and M. R. I. A. 8vo. Callow.

A Syllabus of a Course of Lectures on the Institutes and Practice of Medicine, delivered by Joseph Adams, M. D. F. L. S. 8vo. Callow.

A Syllabus of a Course of Lectures on Pharmaceutic Chemistry, delivered by John Ayrton Paris, M. B. F L. S. 8vo Callow.

A Treatise on the Gout; containing the opinions of the most celebrated ancient and modern Physicians on that disease; and Observations on the Eau Medicinale. By John Ring, Member of the Royal College of Surgeons in London, and of the Medical Societies of London and Paris. 8vo. Callow.

### NOTICES TO CORRESPONDENTS.

We are much obliged to our friend Dr. J. C. Warren, of Boston, New England, for the tracts which he has forwarded to us; and assure him that his future Communications will be highly acceptable.

From the Signature he has adopted, we do not suppose our Correspondent Ἰήτρων Δυσμενὴς expected that we should insert his facetious letter; on another occasion we shall hope to avail ourselves of his classical pen.

We are much indebted to Lincolniensis for the information contained in his note, and shall be most willing to lay before the public any facts on the subject, authenticated by his real name, and expressed in temperate language.

A pressure of matter, either temporary, or having a prior claim, must afford an apology for us to our friendly correspondent, Mr. Knowles, for not inserting his communication this month. In the next Journal it certainly will appear.

As the subject on which W. H. writes has already had considerable discussion, we trust, he will excuse our inserting his note. As he states himself to be a young practitioner, we may, perhaps, be pardoned for recommending to him an investigation of the local circumstances of his residence, which we understand to be in a remote part of the island. These local circumstances will be comprehended in what may be called a medical Topography, and will consist of a Flora of his neighbourhood; its natural history as it regards quadrupeds, birds, fishes, and insects; the atmospherical peculiarities; the waters and soils; the prevalence of endemial and epidemical diseases; the veterinary art and diseases of cattle; empiricism and nostrums peculiar to the place, &c. &c. &c. On any of these subjects we shall be glad to hear from him. We take this opportunity of representing generally to our Correspondents the importance of collecting facts on the preceding subjects, as affording a direct means of elucidating the actual state of medical science throughout the Empire, of improving its practical part, either by correcting errors, promulgating successful modes of treatment, or by bringing under public notice efficacious cuporistic and popular remedies.

Mr. Hume's Observations on the Eau Medicinale d'Husson will appear in our next Number, as will Mr. Tegart's case of Small-Pox, and also histories of some anomalies in that disease, with Remarks by Medicus.

Communications are received from Dr. Dickson, Mr. Smith, Veritas, Mr. Harrup, Mr. Sur, Mr. Davies, Mr. Want, &c. &c. &c.

Mr. Harrold will see his wish complied with in this Journal; and the Editors assure him they will have much satisfaction in giving a place to his further statement of the Case of Newland, with the elucidations that dissection afforded.

